# Self-reported allergies correlate with a worse patient-reported outcome after hip arthroscopy: a matched control study

**DOI:** 10.1093/jhps/hnab022

**Published:** 2021-04-07

**Authors:** Erica L Swartwout, Jacob D Feingold, Joshua I Wright-Chisem, John M Apostolakos, Sacha A Roberts, Anil S Ranawat

**Affiliations:** Department of Orthopedic Surgery, Hospital for Special Surgery, 535 East 70th Street, New York, NY 10021, USA

## Abstract

Patient-reported outcome measures (PROMs) in patients with and without at least one self-reported allergy undergoing hip arthroscopy were compared. Data on 1434 cases were retrospectively reviewed, and 267 patients were identified with at least one self-reported allergy and randomly matched to a control group on a 1:2 ratio. Four PROMs [Modified Harris Hip Score (mHHS), Hip Outcome Score-Activities of Daily Living (HOS-ADL), Hip Outcome Score-Sports (HOS-Sport) and 33-item International Hip Outcome Tool (iHOT-33)] were collected preoperatively, and at 5–11, 12–23 and 24–35 months postoperatively. Significant PROM differences were found 5–11 months postoperative on mHHS (*P* < 0.001), HOS-ADL (*P* = 0.002), HOS-Sport (*P* < 0.001) and iHOT-33 (*P* < 0.001). At 12–23 months postoperative, the allergy cohort had significantly worse scores on mHHS (*P* = 0.002), HOS-ADL (*P* = 0.001), HOS-Sport (*P* < 0.001) and iHOT-33 (*P* < 0.001). They also had significantly worse measures 24–35 months postoperative on mHHS (*P* = 0.019), HOS-Sport (*P* = 0.006) and iHOT-33 (*P* < 0.001). Multivariable logistic regression showed that each additional allergy reported significantly increased the risk of failing to meet the minimal clinically important difference 5–11 months after surgery on mHHS by 1.15 [OR (95% CI): 1.15 (1.03, 1.30), *P* = 0.014], on HOS-ADL by 1.16 [OR (95% CI): 1.16 (1.02, 1.31), *P* = 0.021] and on iHOT-33 by 1.20 [OR (95% CI): 1.20 (1.07, 1.36), *P* = 0.002]. Results suggest self-reported allergies increase the likelihood of a patient-perceived worse outcome after hip arthroscopy. An understanding of this association by the physician is essential during presurgical planning and in the management of postoperative care.

## INTRODUCTION

The utilization of hip arthroscopy as a treatment option for functional hip pain continues to increase across all age groups [1–3]. As these rates continue to rise, it is critical for the clinician to identify preoperative factors that may help predict postoperative outcomes. Many factors that predict surgical outcomes after hip arthroscopy have already been identified, including surgeon experience, surgical technique and grade of osteoarthritis [4–6]; however, additional patient-specific factors may also affect outcomes following hip arthroscopy.

Previous literature reveals a significant association between patient dissatisfaction with medical care and allergic-type symptoms without a discernible etiology [7]. Accumulating evidence in hip and knee arthroplasty has shown that patients with multiple self-reported allergies have significantly lower functional status after arthroplasty [8]. Further, these patients also reported lower preoperative levels of function and experienced less improvement from arthroplasty [8]. We believe that this pattern is not confined solely to hip arthroplasty and that it is reasonable to infer a similar correlation in hip preservation surgery.

The purpose of this study is to compare patient-reported outcome measures (PROMs) between patients undergoing hip arthroscopy for symptomatic femoroacetabular impingement (FAI) with and without at least one self-reported allergy. Our primary hypothesis is that patients with at least one self-reported allergy, when compared to a matched control group, will have significantly lower outcome scores on postoperative PROMs. Further, we also hypothesize that patients with at least one-self reported allergy when compared to a matched control will (i) achieve the minimal clinically important difference (MCID) after arthroscopic treatment of FAI [9] at a significantly smaller rate and (ii) that an increasing number of self-reported allergies will significantly heighten the risk of failure to meet the MCID after hip arthroscopy. While there has been recent literature that has exposed a novel association between surgical outcome following hip arthroscopy and number of self-reported allergies, most of these studies are limited to a single surgeon and are underpowered [10, 11]. We aim to improve upon the design of these studies and seek to provide a more concrete analysis of the relationship between a self-reported allergy and outcome after hip arthroscopy.

## METHODS

Institutional Review Board approval was obtained at our site before the start of this study. This is a single-center study wherein prospectively collected data on patients with nonarthritic hip disease who presented to our institution between 2010 and 2016 was retrospectively reviewed via a separate IRB-approved hospital-wide registry. The registry is a standard of care multi-surgeon database that tracks the clinical and patient-reported outcomes of nonarthritic hip disease managed conservatively and/or surgically. A retrospective review of medical records and radiographs for 1434 hip arthroscopy cases was completed to classify patients according to allergy reporting status. Allergies reported by the patient during clinical visits were entered by medical staff into our institutions electronic medical record (EMR). Common allergies included penicillin, pollen, dust, nuts and other various allergens. The study excluded patients who (i) had staged procedures with <1 year between surgeries, (ii) had previous ipsilateral hip surgery and (iii) had advanced hip OA (defined as Tonnis Grade >2). After application of these parameters, 267 patients (range of 18–35, mean age = 26.3, standard deviation = 5.6) were identified with at least one self-reported allergy. These patients were then randomly matched on a 1:2 ratio by age and sex to a control group composed of patients who underwent hip arthroscopy with no self-reported allergies (*n* = 534, mean age = 25.8, standard deviation = 5.3). For example, for each patient in the study group, there were two patients in the control group who had baseline parameters, such as age and sex, controlled for.

All patients were assessed with four hip-specific PROMs: The Modified Harris Hip Score (mHHS), the Hip Outcome Score-Activities of Daily Living (HOS-ADL), the Hip Outcome Score-Sports (HOS-Sport) and the 33-item International Hip Outcome Tool (iHOT-33). PROMs were collected at baseline before surgery and at three-time points after surgery: 5–11 months, 12–23 months and 24–35 months postoperatively.

Comparing the postoperative outcome scores between cohorts at each time interval enabled assessment of the benefit of hip arthroscopy. By making comparisons to previously published values for the MCID after arthroscopic treatment of FAI [9], we were further able to determine any difference in outcome between cohorts. Additionally, by performing logistic regression analysis, we were able to ascertain whether an increasing number of self-reported allergies put a patient at higher risk of failure to meet the MCID after hip arthroscopy.

### Statistical analysis

Demographics were reported for the two cohorts, and comparisons were made between the cohorts using a *t*-test and chi-square test for continuous and categorical data, respectively (Table I). T-tests were used to compare mean PROM scores at each time interval for the two cohorts (Table II). Frequency and percentage of patients who achieved the MCID for the mHSS, HOS-ADL, HOS-Sport and iHOT-33 are reported (Table III). Chi-square tests were used to compare the percentage of patients meeting the MCID for the four PROMs at each time interval for the two cohorts. Previously published values for the MCID of 8.2, 8.3, 14.5 and 12.1 on mHSS, HOS-ADL, HOS-Sport and iHOT-33, respectively, have been defined as meaningful improvements and were utilized in this study. A multivariable linear regression model controlling for number of reported allergies, age, sex and preoperative PROM score was performed on the primary outcome of failing to meet the MCID (Table IV). This model was performed 12 separate times (at three-time intervals for each of the four PROMs). Analyses were performed using RStudio version 1.2.1335. All *P* values were reported with the level of significance set at *P* < 0.05.

**Table I.** Patient demographics

	Allergy cohort (*n* = 267)	Control group (*n* = 534)	*P*-value
Sex			
Male	125 (46.8%)	245 (45.9%)	0.861
Female	142 (53.2%)	289 (54.1%)	
Age^a^	26.3 ± 5.6	25.8 ± 5.3	0.225

^a^ Mean shown with SD.

**Table II.** Mean PROM scores

	PROM scores						
	Allergy cohort (*n* = 267)			Control group (*n* = 534)			*P*-value (level of significance *P*<0.05)
	*n*	Mean	SD	*n*	Mean	SD	
mHHS							
Baseline	266	61.5	13.7	534	62.6	12.5	0.299
5–11 months	171	78.0	16.6	385	83.1	14.4	**<0.001**
12–23 months	149	79.6	16.1	302	84.5	15.3	**0.002**
24–35 months	94	84.0	14.9	212	88.2	13.0	**0.019**
HOS-ADL							
Baseline	267	72.1	17.2	534	73.4	15.8	0.403
5–11 months	172	86.8	13.3	388	90.5	12.5	**0.002**
12–23 months	150	86.9	16.3	301	91.8	12.1	**0.001**
24–35 months	95	90.4	13.9	212	93.3	9.9	0.077
HOS-Sport							
Baseline	264	51.1	22.4	523	52.5	22.1	0.315
5–11 months	163	68.1	26.2	375	76.8	23.5	**<0.001**
12–23 months	149	72.8	26.8	291	81.5	23.6	**<0.001**
24–35 months	90	80.5	20.5	209	85.1	20.6	**0.006**
iHOT-33							
Baseline	267	39.1	17.2	534	38.9	17.2	0.808
5–11 months	162	65.8	22.7	361	72.8	21.8	**<0.001**
12–23 months	145	67.1	24.9	278	76.0	22.8	**<0.001**
24–35 months	84	72.5	24.2	177	80.5	20.2	**<0.001**

**Table III.** Percent to meet MCID

	*n* (%) Meeting MCID		
	Allergy cohort (*n* = 267)	Control group (*n* = 534)	*P*-value (level of significance *P*<0.05)
mHHS			
5–11 months	66.7% (114/171)	80.8% (311/385)	**<0.001**
12–23 months	69.8% (104/149)	83.8% (253/302)	**<0.001**
24–35 months	78.7% (74/94)	90.6% (192/212)	**0.008**
HOS-ADL			
5–11 months	61.0% (105/172)	71.9% (279/388)	**0.014**
12–23 months	71.3 % (107/150)	77.7% (234/301)	0.169
24–35 months	66.3% (63/95)	83.0% (176/212)	**0.002**
HOS-Sport			
5–11 months	50.3% (82/163)	65.3% (245/375)	**0.001**
12–23 months	63.1% (94/149)	77.0% (224/291)	**0.003**
24–35 months	65.6% (59/90)	81.8% (171/209)	**0.004**
iHOT-33			
5–11 months	69.1% (112/162)	83.1% (300/361)	**<0.001**
12–23 months	75.2% (109/145)	87.4% (243/278)	**0.002**
24–35 months	79.8% (67/84)	91.5% (162/177)	**0.012**

**Table IV.** Multivariable logistic regression: Effect of CUMULATIVE allergies on failure to meet the MCID when controlling for sex, age and preoperative PROM

	OR (95% CI)	*P*-value (level of significance *P*<0.05)
mHHS		
5–11 months	1.15 (1.03, 1.30)	**0.014**
12–23 months	1.18 (1.04, 1.33)	**0.011**
24–35 months	1.15 (0.93, 1.42)	0.190
HOS-ADL		
5–11 months	1.16 (1.02, 1.31)	**0.021**
12–23 months	1.08 (0.95, 1.22)	0.254
24–35 months	1.08 (0.88, 1.32)	0.473
HOS-Sport		
5–11 months	1.12 (1.00, 1.26)	0.057
12–23 months	1.10 (0.97, 1.24)	0.121
24–35 months	1.09 (0.89, 1.33)	0.402
iHOT-33		
5–11 months	1.20 (1.07, 1.36)	**0.002**
12–23 months	1.13 (0.99, 1.29)	0.077
24–35 months	1.18 (0.95, 1.45)	0.129

## RESULTS

Two hundred and sixty-seven patients were identified who underwent hip arthroscopy with at least one self-reported allergy and randomly matched to a control group on a 2:1 ratio according to age and sex. Basic demographic characteristics of both cohorts are reported in Table I.

The allergy cohort had lower preoperative scores on mHHS, HOS-ADL and HOS-Sport and a higher preoperative score on iHOT-33 when compared to the control group. However, none of these differences were significant. At 5–11 months postoperative, the allergy cohort reported significantly lower scores on mHHS (*P* < 0.001), HOS-ADL (*P* < 0.002), HOS-Sport (*P* < 0.001) and iHOT-33 (*P* < 0.001). At 12–23 months postoperative, the allergy cohort reported significantly lower scores on mHHS (*P* < 0.002), HOS-ADL (*P* < 0.001), HOS-Sport (*P* < 0.001) and iHOT-33 (*P* < 0.001). At 24–35 months postoperative, the allergy cohort reported significantly lower scores on mHHS (*P* < 0.019), HOS-Sport (*P* < 0.006) and iHOT-33 (*P* < 0.001). At 24–35 months after surgery, the allergy cohort reported a worse score on HOS-ADL, however, this value was not significant (*P* < 0.077). Comparisons between cohorts preoperative and postoperative PROMs are presented in Table II.

A significantly smaller percentage of the allergy cohort achieved the MCID at 5–11 months postoperative compared to the control group on mHHS (*P* < 0.001), HOS-ADL (*P* < 0.014), HOS-Sport (*P* < 0.001) and iHOT-33 (*P* < 0.001). This analysis at the 12–23 month interval again showed a significantly smaller percentage of the allergy cohort achieved the MCID on mHHS (*P* < 0.001), HOS-Sport (*P* < 0.003) and iHOT-33 (*P* < 0.002). A smaller percentage of the allergy cohort achieved the MCID on HOS-ADL at 12–23 months postoperative, however, this difference was not significant (*P* < 0.169). At 24–35 months postoperative, a significantly smaller percentage of the allergy cohort achieved the MCID compared to the control group on mHHS (*P* < 0.008), HOS-ADL (*P* < 0.002), HOS-Sport (*P* < 0.004) and iHOT-33 (*P* < 0.012). The raw number and percentage of patients to achieve the MCID in each cohort at each interval can be visualized in Table III.

The multivariable logistic regression models demonstrate that reporting an increasing number of allergies puts patients at a higher risk of failing to meet the MCID at early time points postoperatively. At 5–11 months postoperative, each additional allergy reported significantly increased the patients odds of failing to meet the MCID on mHHs by 1.15 [OR (95% CI): 1.15 (1.03, 1.30), *P* = 0.014], on HOS-ADL by 1.16 [OR (95% CI): 1.16 (1.02, 1.31), *P* = 0.021] and on iHOT-33 by 1.20 [OR (95% CI): 1.20 (1.07, 1.36), *P* = 0.002]. At 12–23 months, each additional allergy reported significantly increased the odds of failure to reach the MCID on only mHHS by 1.18 [OR (95% CI): 1.18 (1.04, 1.33), *P* = 0.011]. The remaining tests at the remaining collection points were insignificant. The full results of the twelve multivariable regression models are illustrated in Table IV.

## DISCUSSION

The results of this study support our initial hypothesis: patients with at least one allergy had significantly worse scores across all PROMs at all postoperative points of data collection with the exception of HOS-ADL at 24–35 months where the allergy cohort still reported a lower average score. Statistical analyses also showed that patients with a self-reported allergy achieved the MCID at a significantly smaller rate than the control group. The MCID is defined as the smallest change in outcome score that a patient considers meaningful [9] and is an important metric that enables a clinician to assess whether a patient benefited from a procedure. A significantly smaller percentage of the allergy cohort achieved the MCID on all PROMs at all points of data collection excluding HOS-ADL at 12–23 months postoperative where the difference was insignificant. The difference in outcome scores and rate of meeting the MCID between the allergy cohort and control cohort substantiates our belief that patient-reported allergies can lead to a worse patient-reported outcome after hip arthroscopy.

Multivariable logistic regression models examined the effect of increasing number of self-reported allergies and likelihood of failing to meet the MCID. It showed that on all four PROMs at 5–11 months postoperative each additional allergy a patient-reported significantly increased the risk of failure to meet the MCID. This analysis of the cumulative effect of allergies suggests that patients with multiple self-reported allergies are at an increased risk of failing to meet the MCID in the short term after hip arthroscopy.

Multiple studies have assessed the relationship between patient-reported allergies and joint arthroplasty [8, 12–15]. In previous studies, it has been shown that patients who report allergies have less postoperative satisfaction after hip and knee arthroplasty [8, 12–15]. Some studies have also considered the relationship between patient-reported allergies and hip arthroscopy; however, these studies were single surgeon studies with a small follow-up time and limited sample size [10, 11]. Our study design improves upon their investigations into the novel relationship between self-reported allergies and outcome after hip arthroscopy.

Prior published literature in total joint arthroplasty has suggested psychological factors can influence postoperative satisfaction rates [12, 13, 16]. Patients who feel a sense of compromised physical or mental wellness are more likely to have worse patient-reported outcomes [12, 13, 16]. Further, a correlation between poorer surgical outcomes and patients with symptoms of depression and anxiety [16–18] or those with negative feelings towards surgery [19] has been established in both arthroplasty and arthroscopy. This relationship has been documented in the field of hip preservation surgery as patients with a history of psychiatric illness were 84% more likely to have 2-year postoperative pain following hip arthroscopy compared to patients without such history [20]. Moreover, there have been published associations with false allergy reporting by patients with Axis I psychiatric disorders such as severe depression and other mood disorders [21, 22]. These findings, paired with the findings of our study, suggest that patient-reported allergies function can identify patients at risk for a worse reported outcome after surgery in a similar manner as mental health surveys. However, no analysis or data intake was completed concerning mental wellbeing in either cohort in our study so this cannot be concluded. A physician should take a guarded approach when utilizing mental health surveys, self-reported allergies or personality traits as prognosticators of surgical outcome.

It is important to note that despite the worse PROM scores reported in our study cohort compared to the control group, improvement is still seen from baseline to postoperative PROMs and over two-thirds of the allergy cohort achieved the MCID on a majority of the PROMs. The majority of the allergy cohort meeting MCID is a testament to the efficacy of hip arthroscopy as a treatment method for FAI regardless of whether a patient reports an allergy. Thus, while patients with a self-reported allergy did not benefit as much as the matched control group in this study, they did benefit from the procedure and should be considered for surgical intervention if diagnosis is indicative of FAI refractory to conservative management.

Our study was not without limitations. While our data was collected in a prospective manner, it was evaluated retrospectively. The cohort give to us was robust; however, the age range was limited to 18–35. As such, the generalizability of our findings may be limited. Additionally, PROMs data was collected in only time range values, for instance, 5–11 months postoperative, rather than mean values. Further, it would have been useful when stratifying these patients to control for certain intraoperative procedures, such as the presence or absence of a cam decompression. However, this information was not available for all patients and therefore not included in this study. Moreover, we were able to analyze subjective patient reported data such as PROMs, however, other objective data such as physical exam findings, radiographic results and subsequent surgeries were not available for all patients and therefore not included in this study. BMI and mental health scores are two other parameters that would have been interesting to consider but were also not available. Additionally, allergy data was collected from our institution’s EMR. The data are self-reported by the patients and entered into the EMR by medical personnel. However, from the authors’ personal experience and previously published literature on self-reported allergies [12], it is not infrequent for allergies to be misreported and/or unreported. Despite these limitations, this study provides insight into how patients with multiple self-reported allergies respond to hip arthroscopy when compared to a control group. Further research, specifically prospective studies with a more comprehensive analysis, is needed to better determine if patients with self-reported allergies are predisposed for inferior outcomes after hip arthroscopy.

## CONCLUSION

Statistical findings support our initial hypothesis that patients with at least one self-reported allergy have inferior self-reported outcomes following hip arthroscopy when compared to a control group. Patients with at least one reported allergy experienced a significantly reduced rate of achieving MCID at each time point. Further, patients with multiple reported allergies may be at an increased risk of failure to meet the MCID in the short-term following hip arthroscopy. However, it is worth noting that these patients do still benefit from hip arthroscopy. An understanding of this association on the part of the physician is essential during presurgical planning and in managing postoperative outcomes.
